# 3D Plotting of Calcium Phosphate Cement and Melt Electrowriting of Polycaprolactone Microfibers in One Scaffold: A Hybrid Additive Manufacturing Process

**DOI:** 10.3390/jfb13020075

**Published:** 2022-06-08

**Authors:** David Kilian, Max von Witzleben, Matthew Lanaro, Cynthia S. Wong, Corina Vater, Anja Lode, Mark C. Allenby, Maria A. Woodruff, Michael Gelinsky

**Affiliations:** 1 Centre for Translational Bone, Joint and Soft Tissue Research, Faculty of Medicine and University Hospital Carl Gustav Carus, Technische Universität Dresden (TUD), 01307 Dresden, Germany; david.kilian@tu-dresden.de (D.K.); max.von_witzleben@tu-dresden.de (M.v.W.); corina.vater@uniklinikum-dresden.de (C.V.); anja.lode@tu-dresden.de (A.L.); 2 Science and Engineering Faculty, Queensland University of Technology (QUT), Brisbane, QLD 4000, Australia; matthew@lanaro.com.au (M.L.); c_wong32@hotmail.com (C.S.W.); mark.allenby@qut.edu.au (M.C.A.); mia.woodruff@qut.edu.au (M.A.W.); 3 Institute of Health and Biomedical Innovation, Queensland University of Technology (QUT), Brisbane, QLD 4059, Australia

**Keywords:** biomaterials, melt electrowriting, 3D printing, bone cement, polycaprolactone, multi-material scaffolds

## Abstract

The fabrication of patient-specific scaffolds for bone substitutes is possible through extrusion-based 3D printing of calcium phosphate cements (CPC) which allows the generation of structures with a high degree of customization and interconnected porosity. Given the brittleness of this clinically approved material, the stability of open-porous scaffolds cannot always be secured. Herein, a multi-technological approach allowed the simultaneous combination of CPC printing with melt electrowriting (MEW) of polycaprolactone (PCL) microfibers in an alternating, tunable design in one automated fabrication process. The hybrid CPC+PCL scaffolds with varying CPC strand distance (800–2000 µm) and integrated PCL fibers featured a strong CPC to PCL interface. While no adverse effect on mechanical stiffness was detected by the PCL-supported scaffold design; the microfiber integration led to an improved integrity. The pore distance between CPC strands was gradually increased to identify at which critical CPC porosity the microfibers would have a significant impact on pore bridging behavior and growth of seeded cells. At a CPC strand distance of 1600 µm, after 2 weeks of cultivation, the incorporation of PCL fibers led to pore coverage by a human mesenchymal stem cell line and an elevated proliferation level of murine pre-osteoblasts. The integrated fabrication approach allows versatile design adjustments on different levels.

## 1. Introduction

Additive manufacturing (AM) offers tremendous possibilities for the generation of patient-specific implants and tissue substitutes. With the use of clinical imaging data based on computed tomography (CT) or magnetic resonance imaging (MRI), individual geometries can be incorporated in the design and fabrication of artificial tissue constructs [1,2]. Extrusion-based 3D printing (3D plotting) is a technique increasingly used in biomedical research and clinical applications for the processing of pasty materials, such as biomaterial inks [3] or bioinks.

Calcium phosphate cements (CPC) are promising materials for additively manufactured bone-forming bioscaffolds. In 2014, Lode et al. demonstrated the capability of 3D plotting to fabricate an open-porous scaffold from pasty CPC based on 60% α-tricalcium phosphate and a carrier oil phase [4]. CPC can be stored as a malleable paste which does not require any temperature elevation (as needed for fused deposition modelling) or pH switch for extrusion-based processing and a delayed post-plotting hardening due to the oil-based carrier liquid [4,5]. During its setting phase in an aqueous or humid environment, water replaces the oil component and nanocrystalline hydroxyapatite (HAp) is formed that closely resembles the native bone mineral phase [5,6] and can be resorbed by osteoclasts in vitro and in vivo [7,8]. Furthermore, in this pasty consistency, CPC can be used to generate highly complex 3D shapes with the use of corresponding sacrificial materials [9]. While factors such as biocompatibility, resorbability and mechanical properties are important for creating scaffolds for bone implants, a range of design properties also need to be considered, such as an open-porous architecture [10,11]. An additional challenge in the case of different sizes and architectures of scaffolds is the integrity of the scaffolds during and after implantation, since the CPC material, despite its good biocompatibility and suitable stiffness, is rather brittle once set. As a result, scaffolds tend to fracture, which can cause smaller fragments to dislocate. As an alternative to CPC, synthetic polymers are often studied as a material for bone substitutes, as they show excellent mechanical properties [12,13] and can be processed with fused deposition modelling (FDM) to generate patient-specific implants. Tailored properties and integrated functionalities can be considered applicable, e.g., for advanced bone tissue engineering in cranial defects with hybrid scaffolds based on blends of calcium phosphates and aliphatic polyesters [14]. Integrity and scaffold stability during handling and (post-)implantation are of particular interest. Disadvantages of plain synthetic polymers include lower levels of biocompatibility, osteoinductivity, osteoconductivity and a long degradation time (3–4 years) with its acidic byproducts, as is typical for the most commonly used polymer in medicine, polycaprolactone (PCL) [12,15,16]. Consequently, different combinations of PCL and calcium phosphates have been investigated in the past [17,18,19,20], as has the design of nanocomposite scaffolds based on PCL and HAp [21] that even allowed blending iron-doped HAp with PCL for magnetic scaffold stimulation [22]. Disadvantageously, all combinations contained a high PCL amount. A reduction of the PCL content is desirable to keep acidic byproducts to a minimum and to decrease the degradation time, but this is difficult to achieve without increasing the brittleness of the composite scaffolds. Therefore, an interesting method to achieve this could be a special and rather new form of solution electrospinning (ES), that is melt electrowriting (MEW), which replaces the usage of cytotoxic solvents (as in ES) with a molten material reservoir, mostly molten PCL [23]. An associated increase in viscosity enables the accurate disposition of microfibers (1–100 µm) through jet stabilization using high voltage [24,25].

The obtained scaffolds exhibit a highly defined fiber diameter with an accurate, predictable laydown pattern [26,27]. This can be of clinical significance for various applications towards replacement of cartilage [28], aortic valve [29] or tympanic membrane [30]. However, scaffolds for osteochondral or bone regeneration require volumetric, clinically relevant dimensions and specific mechanical properties that cannot be achieved by MEW only. Therefore, current research has been focusing intensely on the combination of different fabrication techniques with MEW inside one construct [31]. G. Kim et al. have already described a concept of combining different PCL fiber dimensions by rapid prototyping and electrohydrodynamic writing in one hybrid scaffold to influence pore bridging by seeded cells [32]. This particular behavior was later monitored and quantified for plain PCL meshes of varying strand distances, identifying 250 µm as a fiber distance with a rather fast bridging pattern of seeded cells, as analyzed via mathematical modelling by Buenzli et al. [33]. Xie and colleagues showed that adhesion pattern and proliferation of cells is not only dependent on the pore size, but can also be controlled by varying the fiber orientation in the PCL mesh, thus offering another design opportunity in scaffold architecture [34].

The combination of MEW and extrusion printing in an alternating setup is challenging as the MEW process requires a homogenous electrical field (E-field) for exact fiber deposition, this is significantly deteriorated when objectives, e.g., CPC strands, are present and create an inhomogeneous E-field [35]. In previous works, MEW-PCL fibers and CPC were combined when modeling tissue transitions, such as periodontal tissue [36] and the cartilage-bone boundary [37]. In both cases, the MEW meshes mimicked the softer tissue and were printed in advance of the other materials to avoid the inhomogeneity of the electric field.

In order to develop a versatile approach for open-porous hybrid CPC+PCL scaffolds, the fundamental aim of our study was to combine two techniques, 3D plotting and MEW, into one hybrid fabrication process with a flexible, alternating pattern to generate stable hybrid scaffolds with the two materials in an interconnected architecture for the design and fabrication of bone substitutes. This involved the application of MEW over extruded, 3D plotted strands of CPC inside a high voltage field. The challenge of this strategy was to stack CPC strands with a high shape fidelity in several layers while concurrently ensuring accuracy of the PCL microfiber formation at the CPC–PCL interface and inside CPC macro-pores. The microstructure of the CPC–PCL interface, as well as the integrity of the hybrid system was evaluated. We were able to produce scaffolds with a clinically relevant height of 3 cm. Additionally, the biological and mechanical responses on various CPC+PCL scaffold architecture were studied, exhibiting that the PCL fibers hold the CPC fragments in place upon scaffold failure under mechanical load. Hence, the robustness of the CPC scaffolds can be improved with a small fraction of PCL. In addition, we increased the CPC strand distance to investigate the scaffold quality/shape fidelity of MEW fibers, and the impact that CPC strand distance had on bridging behavior of murine calvarial osteoblast precursor cells (MC3T3-E1) and immortalized human mesenchymal stem cells (hTERT-MSC) over 2 weeks. We hypothesized, that the incorporation of PCL microfibers will support the seeding efficiency and bridging effect of seeded cells above a certain critical CPC strand distance.

## 2. Materials and Methods

### 2.1. Material Preparation and One-Process Scaffold Fabrication

Calcium phosphate cement CPC (Plotter Paste-CPC, INNOTERE GmbH, Radebeul, Germany) and polycaprolactone PCL Purasorb^®^ PC12 (Corbion Purac, Amsterdam, The Netherlands; CAS 24980-41-4) with a molecular weight of 80 kDa [38] were used for scaffold fabrication. CPC and CPC+PCL scaffolds were generated in a one-step process with an interchangeable pattern using the open-source *Biofabricator*, a customized, multi-purpose AM device developed and housed at the Science and Engineering Faculty, Queensland University of Technology (QUT), and BioScaffolder 3.1 (GeSiM, Radeberg, Germany) [39]. Melt electrowriting (MEW) uses molten thermoplastics to process them with high voltage into micrometer-thin fibers that can be precisely deposited. 3D plotting (3D extrusion printing, 3DP) often uses air pressure to extrude a paste or hydrogel out of a cartridge without thermal treatment. For the production of the scaffolds, the printing processes were alternated layer by layer. MEW was operated with a high voltage field of 8.1 kV and a PCL temperature of 73 °C with a mean dosing pressure of 20 kPa. Printing velocity was chosen at 10 mm s^−1^ while the distance between the nozzle (Ø 250 µm) and the respective scaffold height was kept constant at 5 mm. These parameters allowed printing of uniform fibers of 20 µm, when printed without the presence of CPC, and with a fiber spacing of 250 µm. For CPC extrusion printing (3DP), a conical nozzle with a tip diameter of 410 µm was used at a pressure of 70 kPa and a velocity of 7 mm s^−1^. All scaffolds were printed on glass plates.

Combined scaffolds for cell culture experiments consisted of two perpendicular, meandering, open-porous layers of CPC with two perpendicular layers of PCL written in between. CPC scaffold dimensions were ca. 5 mm × 5 mm × 2 layers, while X and Y for varying the CPC strand distances were slightly adjusted to allow isoform CPC pore sizes over the scaffolds. CPC strand distance was defined as the distance from the respective middle of each strand to the middle of the parallelly aligned neighboring strands. To fit the PCL scaffolds dimensions to those of the CPC scaffold, they were programmed larger, ca. 7 mm × 7 mm × 2 layers due to the local offset (“lag”) between nozzle and deposition [27].

**Figure 1:**
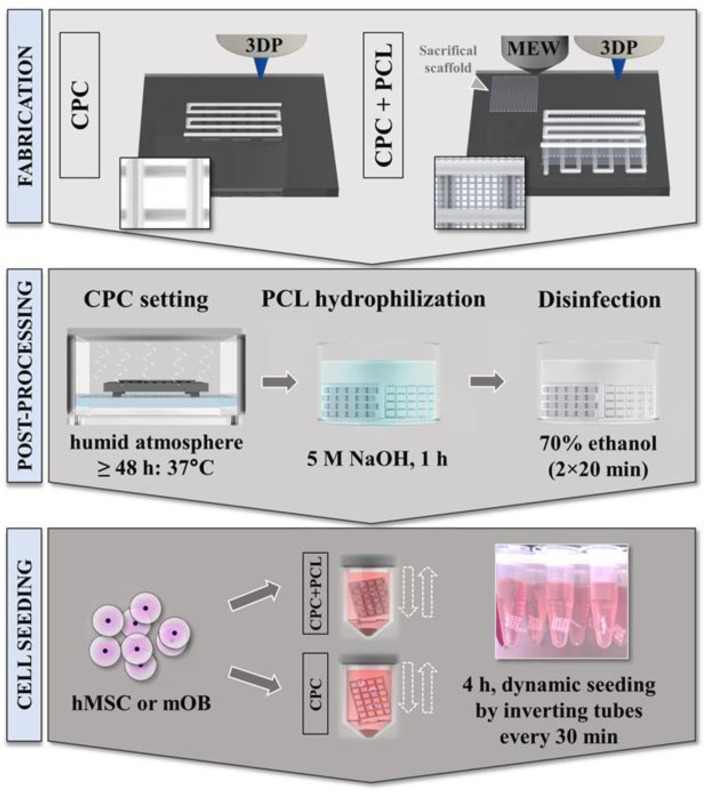
Schematic illustration of the one-step hybrid bioscaffolding process based on MEW and 3D plotting (3DP; **upper panel**) in an alternating pattern, followed by post-processing of the scaffolds through CPC hardening in humid atmosphere, PCL hydrophilization and disinfection (**middle panel**) for the in vitro cell seeding experiments (**lower panel**).

For combined scaffolds for mechanical analysis, a minimum scaffold height of 2.5 mm was required for reliable measurements during compressive deformation. Therefore, ten CPC-layers (10 × 10 mm^2^) were printed with a mesh of two PCL-layers (12 × 12 mm^2^) between the CPC layers, hence nine PCL meshes in total. This led to an overall printing time of ca. 3 h per scaffold, mainly due to the initialization process of the MEW unit. Each time the cartridge was switched to MEW after a 3D-plotted CPC layer, a sacrificial PCL scaffold (Figure 1) was printed (and discarded later) to obtain a uniform fiber diameter and to avoid long beading occurrence, a phenomenon described previously [40]. CPC hardening was induced by keeping the scaffolds in a humid atmosphere and at a constant temperature of 37 °C for >48 h.

Scaffold structure, CPC to PCL orientation and microfiber accuracy before and after hardening were monitored and qualitatively evaluated using a complementary metal oxide semiconductor (CMOS) microscope camera (ProSciTech, Kirwan, QLD, Australia) and a stereo microscope M205 C equipped with a DFC295 camera (Leica Microsystems GmbH, Wetzlar, Germany).

A more complex construct of CPC+PCL for demonstration purposes was designed and fabricated with maximum dimensions of 25.7 mm × 22.4 mm, with six CPC layers and a total of 10 PCL microfiber layers (2 microfiber meshes fabricated between each of the CPC layers). The structure was observed using a light microscope Olympus SZX16 (Olympus Europa SE & Co. KG, Hamburg, Germany).

### 2.2. Scanning Electron Microscopy of CPC+PCL Scaffolds with and without Seeded Cells

CPC and CPC+PCL scaffolds before and after cell seeding and incubation for up to 14 days were imaged via a scanning electron microscope JEOL 7001F (Jeol Ltd., Akishima, Japan) operated at a voltage of 5 kV in a working distance of 8–12 mm, provided by the Science and Engineering Faculty, QUT. For sample preparation, scaffold samples were fixed with 4% formaldehyde (CAS 50-00-0) and glutaraldehyde (CAS 111-30-8) and dehydrated in a PELCO BioWave^®^ Pro+ (Ted Pella Inc., Redding, CA, USA) using a PELCO Microwave Microcentrifuge Tubes PTFE Holder. In brief, samples were vacuum dried in a series of increasing ethanol concentrations (30–100%; CAS 64-17-5), with additional dimethyl sulfoxide (DMSO; CAS 67-68-5) treatment. Samples were coated with a gold layer using a sputter coater EM SCD005 (Leica Microsystems GmbH, Wetzlar, Germany) at 30 mA for 90 s.

### 2.3. Mechanical Characterization of CPC and Hybrid CPC+PCL Scaffolds

Uniaxial compressive testing was applied to 3D plotted CPC scaffolds (dimensions ca. 10 × 10 × (2.5–3.0) mm^3^, 10 CPC layers) with and without integrated PCL fibers (250 µm fiber spacing and 12 × 12 mm^2^, 9 × 2 PCL layers) after NaOH (60 min, 5 M) treatment using the universal testing machine Zwick-Roell Z010 (ZwickRoell, Ulm, Germany) equipped with a 10 kN load cell. Evaluation of the Young’s modulus *E* was carried out at the first two linear slopes in the obtained stress–strain curves. The compressive strength *F* was measured at the local maximum value (breaking point) of these linear slopes (Supplementary Material, Figure S1). Stability of the CPC to PCL interface and integrity in a CPC+PCL scaffold (dimensions: 10 × 10 × 3 mm^3^, 10 CPC layers with 9 × 2 PCL layers (18 × 18 mm^2^) was qualitatively investigated by applying a tensile force to the outer PCL fibers while the PCL fibers at the opposing scaffold side were attached to a sample holder.

### 2.4. Scaffold Preparation for In Vitro Studies

For hydrophilization of the PCL fibers, all scaffold types (CPC, CPC+PCL) were placed in 5 M NaOH etching solution for 60 min. Afterwards, scaffolds were submerged in 70% ethanol for 20 min twice as a disinfection step (Figure 1, middle panel). Scaffolds were then kept in αMEM (ThermoFisher, Waltham, MA, USA) with 1% penicillin/streptomycin at 37 °C and under 5% CO_2_ overnight for equilibration, followed by another washing step in respective cell culture medium (αMEM/DMEM) for 10 min to remove residues of hydrophilization/disinfection.

Scaffolds for the first presented seeding experiment with scaffold designs CPC_800_+PCL and CPC_1200_+PCL were fabricated with respective experiments performed at QUT, in combination with scanning electron microscopical analysis of cell-seeded and cell-free scaffolds. Other in vitro experiments and material characterization were conducted at Technische Universität Dresden (TUD).

### 2.5. Cell Cultivation and Scaffold Seeding Procedure

Two different cell types, a murine calvarial osteoblast precursor cell line (mOB, MC3T3-E1, #ACC210, Leibniz-Institut DSMZ, Braunschweig Germany) and an immortalized human mesenchymal stem cell (hMSC) line expressing human telomerase reverse transcriptase (hTERT, hTERT-MSC) were used [41]. The hTERT-MSC were kindly provided by Prof. Matthias Schieker (Laboratory of Experimental Surgery and Regenerative Medicine, University Hospital Munich (LMU), Munich, Germany). For expansion/cultivation of mOB, αMEM with 9% FCS and 1% pen/strep was used, while during expansion/cultivation of hMSC, DMEM with 9% FCS and 1% pen/strep was applied. Scaffolds were seeded dynamically (tube inversion every 30 min) for 4 h in 1.5 mL Eppendorf tubes filled with 1 mL of cell suspension (1.0 × 10^5^ cells) (Figure 1, lower panel). Varying CPC strand distances (800–1600 µm) were chosen for in vitro experiments. To consider potentially irregular pore shapes, CPC pore diameters were determined as the maximum diagonal size of the pore measured using Fiji ImageJ 1.51 h.

### 2.6. Analyzing Cell Distribution and Pore Bridging Behavior

By gradually increasing the CPC strand distance in scaffolds, the impact of porosity on the pore bridging behavior of cells was evaluated. A critical value of CPC strand distance might be determined for the size of pores that cannot be fully colonized by bridging of cells without PCL microfibers, but can be after addition of the PCL microfibers. On Days 1, 3, 7 and 14 after seeding, cell distribution was analyzed after cytoskeletal and nuclear fluorescence staining. Cell-seeded scaffolds were fixed in 4% formaldehyde in PBS for 45 min after washing them in PBS. Permeabilization of the cell membrane was achieved by 5 min incubation in 0.1% Triton-X100/PBS. To avoid unspecific binding, an additional blocking step of 60 min using 1% bovine serum albumin (BSA, Carl Roth GmbH + Co. KG, Karlsruhe, Germany; CAS 9048-46-8) in PBS was performed. Afterwards, cellular nuclei and cytoskeletons were stained with 1.0 µg·mL^−1^ DAPI (Life Technologies, Carlsbad, CA, USA) and 0.17 µM Phalloidin AlexaFluor 488 (ThermoFisher, Waltham, MA, USA). For the in vitro experiment comparing CPC strand distances of 800 and 1200 µm, the fluorescence imaging system Leica AF6000X (Leica Microsystems GmbH, Wetzlar, Germany) was used for observing z-stacks with a mean thickness of 300–500 µm. For the other experiments comparing two different cell types, a Keyence BZ-X810 fluorescence imaging system (Keyence Deutschland GmbH, Neu-Isenburg, Germany) was used, operated at magnification 10× and 4× (z-stacks of a thickness 60–170 µm).

Additionally, scaffolds were stained with a recombinant Alexa Fluor 647-tagged Anti-Vinculin antibody (anti-human; host: rabbit; dilution 1:100 in 1% BSA) by incubation for 2 h at RT, in order to observe focal adhesion points of hMSC to CPC+PCL scaffolds. Scaffolds were imaged using a confocal laser scanning microscope (cLSM; SP5, Leica Microsystems GmbH, Wetzlar, Germany). Excitation and emission settings of photomultiplier tubes (PMT) and hybrid detectors (HyD) were chosen accordingly (DAPI: excitation 405 nm, emission 415–478 nm; phalloidin: ex 488 nm, em 498–560 nm; vinculin: ex 633 nm, em 640–710 nm), with collected z-stack sizes ranging from 40 to 200 µm.

### 2.7. Cell Number Analysis in CPC and CPC+PCL Scaffolds with Varying CPC Strand Distance

On days 1, 3, 6 and 14, cell-seeded samples were collected and washed in PBS for 5 min and placed in −80 °C until required. DNA content in the scaffolds after cell lysis by incubating scaffolds for 4 h at 4 °C in 1 % Triton-X100 in PBS was analyzed by Quant-iT™ PicoGreen™ dsDNA Assay-Kit (Invitrogen/ThermoFisher, Waltham, MA, USA) and Quantifluor assay (dsDNA Assay, Promega, Madison, Wisconsin, USA) according to the manufacturers’ protocols. (Relative) cell numbers per scaffold were calculated via linear regression using a calibration curve of defined numbers of cells (5.0 × 10^3^–2.0 × 10^6^) collected and stored at −80 °C at the day of scaffold seeding.

### 2.8. Statistical Analysis

Statistical analysis of replicate experiments was performed using GraphPad Prism 8. One-way ANOVA and two-way ANOVA with Bonferroni correction were applied. For experiments comparing two experimental groups only, the unpaired *t*-test with Welch correction was chosen accordingly. Evaluating statistical significance, a confidence interval of 95% with *p* < 0.05 was considered.

## 3. Results

### 3.1. Successful Fabrication of Hybrid Scaffolds by One Printing Process Combining 3D Plotting and Melt Electrowriting

**Figure 2:**
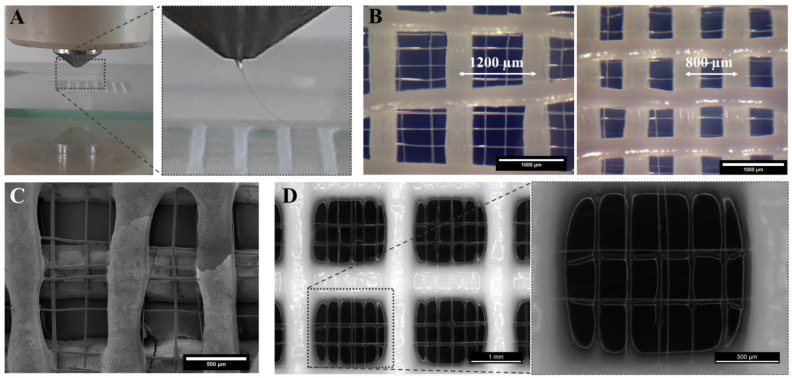
(**A**) Hybrid CPC+PCL scaffold fabrication: MEW process over the first layer of 3D plotted CPC strands on a glass plate. (**B**) Light microscope images of incorporated PCL microfibers between two perpendicular CPC layers in scaffolds with a mean CPC strand spacing of 1200 µm (**left**) and 800 µm (**right**), prior to initiation of the setting reaction in humid atmosphere, scale bar = 1000 µm. (**C**) SEM image of a CPC+PCL scaffold with integrated PCL fibers getting drawn towards the parallel CPC strands of the first/previous layer below, scale bar = 500 µm. (**D**) Stereo light microscope images of CPC_2000_+PCL with a CPC layer (strand distance = 2000 µm) below and above the PCL mesh, prior to post-processing; scaffold design overview (scale bar = 1 mm) and PCL microfibers in one CPC macropore (right, scale bar = 500 µm).

Combining the two fabrication methods of 3D plotting and MEW, as demonstrated in Figure 2A, resulted in CPC and hybrid CPC+PCL scaffolds of a high shape fidelity with a PCL mesh integrated in between two consecutive, perpendicular CPC layers in open-porous CPC scaffolds according to a qualitative visual evaluation (Figure 2). PCL microfibers were placed by MEW onto the first layer of 3D plotted CPC (Figure 2A) with the parallel PCL fibers experiencing some dragging effect towards the parallel CPC strands beneath, as clearly visible via SEM imaging (Figure 2C). However, the pre-defined 90° orientation of both CPC strands and PCL fibers remained intact. This successful combination of the two materials into one integrated hybrid scaffold provided the basis for further microstructure assessment, biological investigation on proliferation and pore bridging behavior, as well as mechanical characterization of hybrid scaffolds. In these experiments, several scaffold architectures with varying CPC strand distances (CPC_x_) of x = 800 µm, 1200 µm (Figure 2B), 1400 µm, 1600 µm and 2000 µm (Figure 2D) were compared.

**Figure 3:**
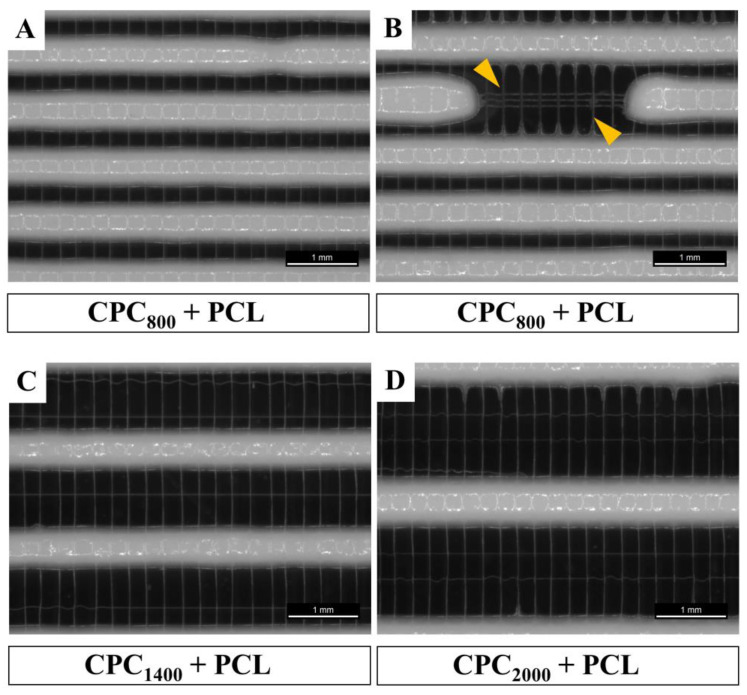
Stereo-light microscope images of PCL meshes (distance 250 µm) printed over 3D plotted CPC strands of different strand distances ((**A**,**B**) 800 µm, (**C**) 1400 µm, (**D**) 2000 µm). (**B**) Position of a CPC strand with interrupted extrusion (off-print for demonstration purposes only which was not applied in further experiments) to illustrate the PCL fibers getting drawn towards CPC strands in parallel orientation (yellow arrows), scale bars = 1 mm.

Comparing the resulting MEW fiber laydown patterns onto varying CPC strand distances (800–2000 µm) in the first CPC layer with a MEW mesh of two perpendicular layers placed on top, we observed that in the fiber direction perpendicular to the first CPC strand layer, the fibers were placed in an accurately adjusted fashion with a homogeneous distance of 250 µm, whereas applying MEW in parallel to the CPC strand underneath resulted in fiber dragging towards the CPC strands. This is exemplarily shown for three different CPC strand distances in the desired range 800–2000 µm in Figure 3.

### 3.2. Microstructure of CPC+PCL Scaffolds after NaOH Treatment

**Figure 4:**
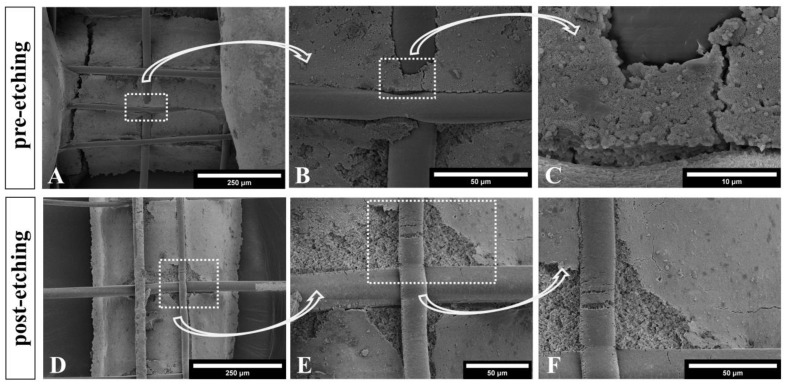
Scanning electron microscopy images of NaOH treated (5 M, 60 min) samples for hydrophilization of the PCL inside hybrid CPC+PCL scaffold for cell attachment (**D**–**F**), and a non-treated CPC+PCL scaffold (**A**–**C**). Both samples reveal formation and maintenance of nanocrystalline hydroxyapatite structures. Scale bars represent 250 µm in (**A**,**D**), 50 µm in (**B**,**E**,**F**) and 10 µm in (**C**).

To enhance cell-adhesive properties of the PCL for the in vitro investigation, hydrophilization using 5 M NaOH was applied after completing the CPC setting reaction. This etching solution was added to both scaffold types with and without PCL fibers. The microstructure of the CPC to PCL interface was examined in etched and non-etched scaffolds via SEM observation (Figure 4). NaOH treatment clearly resulted in a rougher surface texture of PCL fibers, which also plays a role in cell adhesion. Some small cracks in PCL were visible after etching treatment, possibly due to residual stress in the fiber upon quenching during deposition and setting reaction of the CPC.

Additionally, the CPC phase of the brittle scaffolds was slightly damaged through scaffold processing. However, no obvious influence on the CPC microstructure was observed after the 60 min 5 M NaOH treatment. The typical nanocrystalline structure of HAp remained intact (Figure 4C,F).

### 3.3. Mechanical Properties of Hybrid CPC+PCL Scaffolds

**Figure 5:**
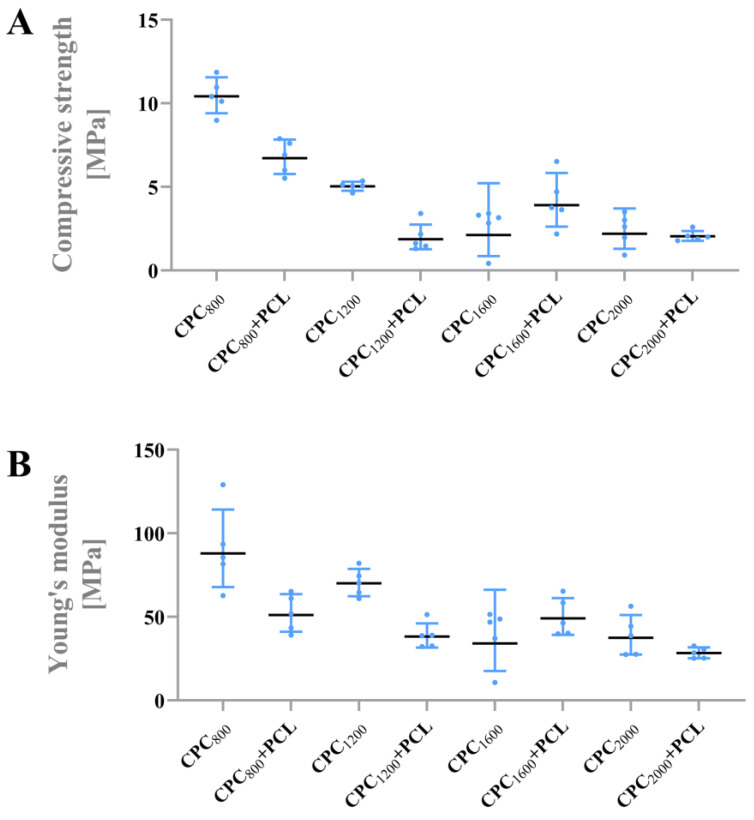
Mechanical properties of CPC and CPC+PCL scaffolds with different CPC strand distances (800 µm, 1200 µm, 1600 µm, 2000 µm) (**A**) Compressive strength of CPC and CPC+PCL scaffolds. (**B**) Young’s modulus of CPC and CPC+PCL scaffolds. (n = 5).

During intensive compressive testing of varying CPC+PCL scaffold designs, two breaking points were observed in the scaffolds’ stress–strain curves. According to the stress–strain curves (representative curve of a CPC_800_+PCL scaffold shown in Supplementary Material, Figure S1), we analyzed compressive strength and Young’s modulus for plain CPC scaffolds and CPC+PCL scaffolds with an open-porous structure and CPC strand distances of 800, 1200, 1600 and 2000 µm (Figure 5). Compressive strength and Young’s modulus were determined respectively for the first breaking point at lower stress and are depicted in Figure 5.

An expected trend of a decreasing compressive strength (Figure 5A) and Young’s modulus (Figure 5B) with increasing CPC strand distance was observed. In most conditions, the addition of PCL microfibers resulted in slightly decreased compressive strength and Young’s modulus. The effect appeared more drastic at the first breaking point in the stress–strain curve of scaffolds with smaller CPC strand distances. Combination of PCL microfibers with CPC scaffolds, with a strand distance of 1600 µm, resulted in a higher mean value for compressive strength and Young’s modulus. For CPC_800_ and CPC_1600_, the standard deviation appeared smaller after addition of PCL microfibers.

After persistently applying load after the first fracture, compressive strength and Young’s modulus were additionally analyzed using the second breaking point of the stress–strain curve (Supplementary Material, Figure S2) in order to identify possible differences between composite structures and pure CPC scaffolds in response to an additional scaffold fracture. For a few of the CPC_2000_+PCL scaffolds, no second breaking point in the stress–strain curve was detected. Therefore, it was not considered in the respective graphs.

### 3.4. Improved Integrity of Fiber-Reinforced Scaffolds

**Figure 6:**
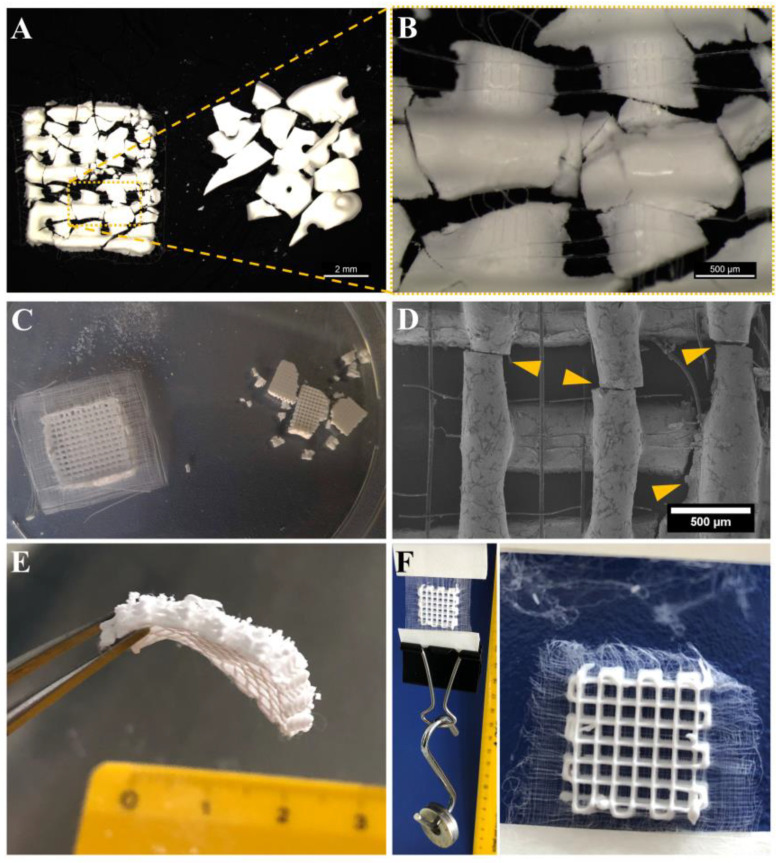
Increasing scaffold integrity in open-porous CPC+PCL scaffolds for clinical application. (**A**) Micrograph illustrating integrity of a CPC+PCL (**left**) and a monophasic CPC (**right**) scaffold (5 mm × 5 mm × 2 CPC layers) after applying mechanical force on brittle CPC structure, scale bar = 2 mm. (**B**) PCL mesh holding together pieces of damaged CPC strands, scale bar = 500 µm. (**C**) Volumetric scaffolds of larger dimensions (10 × 10 × 3 mm³, 10 CPC layers with 9 × 2 PCL layers (18 × 18 mm²), after compressive testing. (**D**) SEM of PCL fibers ensuring integrity in the case of partly damaged (yellow arrows) CPC strands in cell-seeded hybrid CPC+PCL scaffolds (hOB, day 1), scale bar = 500 µm. (**E**) Addition of PCL microfibers allowed bending of a scaffold after occurrence of cracks in the brittle CPC, scale in cm. (**F**) Photograph of a weight of 36 g attached to the PCL mesh inside a scaffold (left, 10 × 10 × 3 mm^3^, 10 CPC-layers with 9 × 2 PCL layers (18 × 18 mm²), CPC strand distance 2.0 mm) and tearing of the PCL fibers outside the CPC+PCL scaffold zone with higher tension by increasing weight, scale in cm.

Comparing the integrity and stability of the CPC and CPC+PCL scaffolds after harsh handling and after defined mechanical testing, photographs in Figure 6 clearly revealed the positive effect of PCL microfibers on the scaffolds. In a PCL-free scaffold, the brittle CPC lost its integrity entirely upon strand breakage (Figure 6A,C, right), while by scaffold support through PCL microfibers, the integrity of the overall scaffold was maintained (Figure 6A,C, left) so that no macro-scaled CPC residues or scaffold fragments could separate from the scaffold after fracture (Figure 6B,D). This also allowed bending and deforming of the scaffold after the occurrence of CPC fractures (Figure 6E) which is otherwise not possible with the brittle CPC scaffold.

For testing the stability of the CPC–PCL interfaces, a PCL frame of a hybrid scaffold was attached to two tape strips above and below, while weight was added below. The scaffold remained stable with a weight of 36 g added at the bottom, which corresponds to 11.8 kPa. By increasing the weight further, fiber network in the PCL frame ruptured without the PCL fibers being dragged out of the CPC scaffold (Figure 6F), indicating the stability of the interconnected CPC+PCL network.

### 3.5. Seeding Efficiency, Proliferation and Pore Bridging Behavior of mOB in CPC and CPC+PCL Scaffolds of Low CPC Strand Spacing (800 µm vs. 1200 µm)

As observed via SEM of cell-seeded CPC+PCL scaffolds, several mOB at the interface of the two materials attached to both material phases forming the basis for a pore-bridging network (Supplementary Material, Figure S3). In the case of loose PCL fibers by a larger frame around the CPC scaffold, cells appeared to attach to both materials equally (Supplementary Material, Figure S4). Based on these findings, for the following in vitro experiments, CPC and CPC+PCL scaffolds of varying designs seeded with two different cell types (mOB and hMSC) were compared. The CPC strand distance was gradually increased from 800 µm to 1200 µm and 1600 µm to 2000 µm. Up to a strand distance of 1600 µm, stable PCL microfibers were found after fabrication. The resulting maximum diagonal CPC pore diameters are summarized in Table 1. For experiment #2 with CPC+PCL_1200_ scaffolds, an altered CPC strand thickness led to a slightly decreased pore diameter (591 ± 82 µm) compared to experiment #1 (685 ± 72 µm).

**Figure 7:**
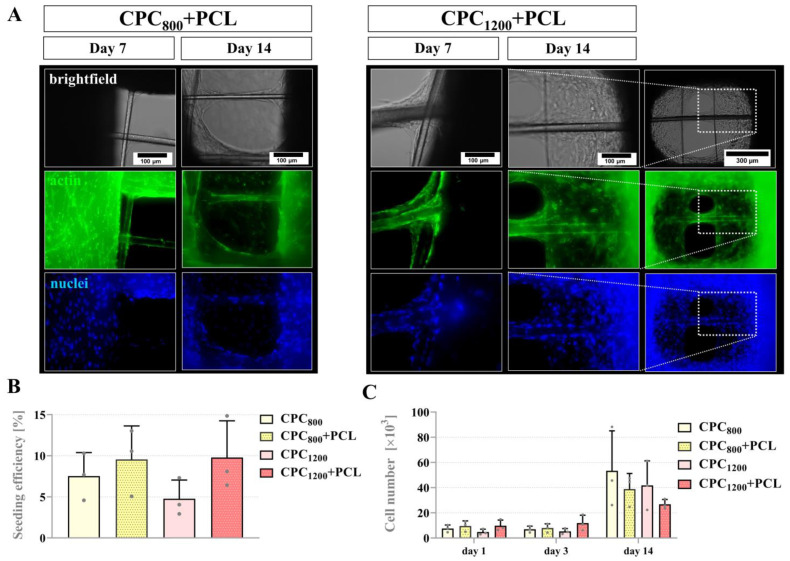
(**A**) Fluorescence images of mOB cell distribution and morphology on day 7 and 14 of cultivation, brightfield image of the scaffold and cell layer in an open-porous CPC+PCL scaffold (**left**: CPC spacing 800 µm, **right**: CPC spacing 1200 µm), DAPI-stained cell nuclei (blue), Phalloidin-stained cytoskeleton (green), scale bar = 100 µm (right column day 14, scale bar = 300 µm). (**B**) Seeding efficiency of mOB (after DNA assay, in % of the initially applied total cell number 10^5^) on different strand spacing patterns of CPC and CPC+PCL scaffolds, mean ± SD, n = 3, differences not statistically significant for *p* < 0.05 (n. s.) (**C**) mOB cell numbers per scaffold at day 1, 7 and 14 of cultivation, mean ± SD, n = 3, n. s.

An initial in vitro experiment was performed with one cell type (mOB) in four different scaffold conditions with a CPC strand distance of 800 and 1200 µm, with and without incorporated PCL microfibers (Figure 7A). In all observed conditions (including plain CPC scaffolds), cells were able to cover large areas of the pores after 14 days. Quantification of cell numbers on scaffolds by DNA assay revealed no significant differences when comparing the cell numbers per scaffold on day 1 (seeding efficiency in % of initially applied cell number 10^5^, Figure 7B) and proliferation over time (Figure 7C) for all scaffolds with and without PCL microfibers, even independent of their CPC strand density and, therefore, surface area.

### 3.6. Seeding Efficiency, Proliferation and Bridging Behavior of mOB and hMSC in CPC and CPC+PCL Scaffolds with CPC Pore Diameter < 800 µm

**Figure 8:**
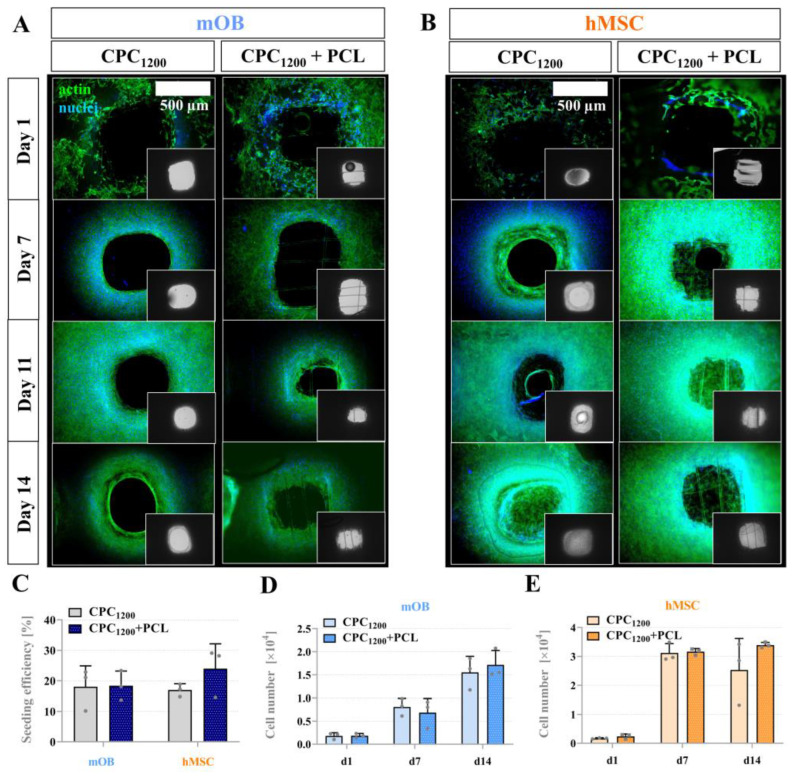
(**A**) Fluorescence images of mOB cell distribution, DAPI-stained cell nuclei, phalloidin-stained actin filaments of the cytoskeleton (**insets**: brightfield image of scaffold position), day 1, 7, 11 and 14; scale bar = 500 µm (**B**) Fluorescence images of hMSC cell morphology, DAPI-stained cell nuclei, phalloidin-stained actin filaments of the cytoskeleton (**insets**: brightfield image of scaffold position), day 1, 7, 11 and14; (**C**) Cell seeding efficiency (after DNA assay, in % of the initially applied total cell number 10^5^) for both cell types mOB and hMSC in CPC and CPC+PCL scaffolds, calculated as the ratio of cells detected via DNA assay from samples on day 1 and the number of totally seeded cells via cell suspension (1 × 10^5^ cells), n = 3, mean ± SD, n. s., (**D**) mOB cell numbers per scaffold at day 1, 7 and 14 of cultivation, n = 3, n. s. (**E**) hMSC cell numbers at day 1, 7 and 14 of cultivation, n = 3, n. s.

All scaffold types could feasibly support viable long-term mOB proliferation. To investigate differences in scaffold-mediated pore bridging across murine and human models, we examined pore bridging kinetics of CPC_1200_ and CPC_1600,_ with or without PCL, with mOB and hMSC. Figure 8 illustrates the cell adhesion of mOB and hMSC over a course of 2 weeks with nuclear and cytoskeletal fluorescence staining via fluorescence microscope imaging. In this experiment, the CPC mean strand distance of 1200 µm resulted in pore distances of 591 ± 82 µm as the maximum distance to be bridged. As shown in Figure 8A, mOB were not able to entirely bridge the pores with and without PCL after 2 weeks, while full pore bridging was achieved by hMSC between day 7 and day 11 for CPC+PCL scaffolds, and for plain CPC scaffolds between day 11 and day 14 of the experiment (Figure 8B). However, no significant differences were reflected in the quantitative analysis of seeding efficiency (Figure 8C) for the comparison of CPC and CPC+PCL scaffolds for both cell types at day 1 after seeding and for cell number development over time (Figure 8D,E). The hMSC did not proliferate further after day 7, which might indicate that the maximum cell density for the scaffold area was reached for both CPC and CPC+PCL. The cell density visible at the outer edges of the images on CPC strands depended on the z-stack selected (z > 60 µm) during imaging and did not reflect the absence of cells there.

### 3.7. Proliferation and Bridging Behavior of hMSC and mOB Affected by PCL Microfibers in Scaffolds with a CPC Pore Diameter > 1300 µm

**Figure 9:**
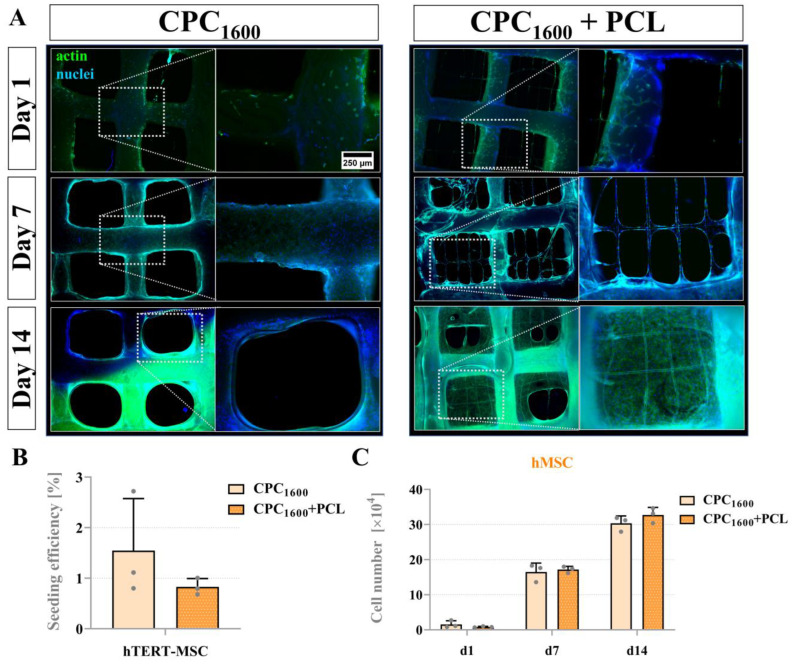
(**A**) Fluorescence images of hMSC cell morphology on day 1, 7 and 14 of cultivation in CPC and CPC+PCL scaffolds with a CPC strand distance of 1600 µm. green = phalloidin, blue = DAPI, scale bar = 500 µm in overview image, scale bar = 250 µm in strand close-up. (**B**) hMSC seeding efficiency (after DNA assay, in % of the initially applied total cell number 10^5^) on CPC and CPC+PCL, mean ± SD, n = 3, n. s. (**C**) hMSC cell numbers per scaffold after 1, 7 and 14 days of cultivation, mean ± SD, n = 3, n. s.

By further increasing the mean strand distance of CPC strands to 1600 µm, a significant difference in the bridging behavior of hMSC comparing CPC and CPC+PCL was observed. Figure 9A illustrates the location of the cells and cellular networks on CPC, CPC+PCL and on PCL microfibers inside the CPC macro-pores. Whereas in plain CPC scaffolds (left), no bridging effect was observed, cells in CPC+PCL were able to bridge the entire CPC pore by day 14. Quantification revealed no significant differences after seeding (day 1, Figure 9B) and after comparing the number of proliferating cells per scaffold over time (Figure 9C).

By cLSM imaging, the morphology of hMSC during pore bridging was observed over time, along with the formation of focal adhesion points (by vinculin staining) in different magnification. We noticed that both the density of cytoskeleton (by phalloidin staining, Supplementary Material, Figure S5, green) and the expression of vinculin as the main component of focal adhesion increased over time from day 7 to day 14 (Supplementary Material, Figure S5).

**Figure 10:**
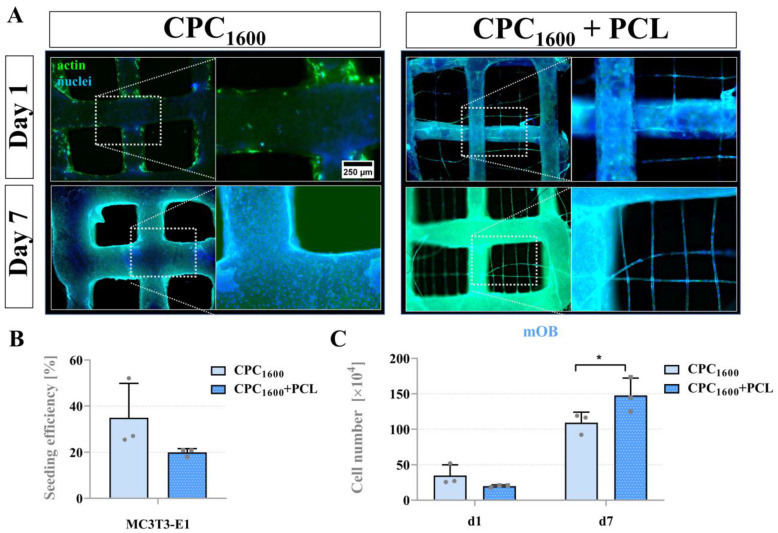
(**A**) Fluorescence images of mOB cell morphology on day 1 and 7 of cultivation in CPC and CPC+PCL scaffolds with a CPC strand distance of 1600 µm. green = phalloidin, blue = DAPI, scale bar = 500 µm in overview image, scale bar = 250 µm in strand close-up. (**B**) mOB seeding efficiency (after DNA assay, in % of the initially applied total cell number 10^5^) on CPC and CPC+PCL, mean ± SD, n = 3, n. s. (**C**) mOB cell numbers per scaffold after 1 and 7 days of cultivation, mean ± SD, n = 3, * *p* < 0.05.

While the hMSC cell line showed a clearly visible difference in bridging behavior observed via fluorescence microscopy, no pore-bridging effects by mOB were observed until day 7 (Figure 10A). Quantitative analysis revealed that a significantly elevated number of cells per scaffold were found on the CPC+PCL scaffolds compared to plain CPC (Figure 10C).

### 3.8. Design and Fabrication of a Complex CPC+PCL Construct Geometry

**Figure 11:**
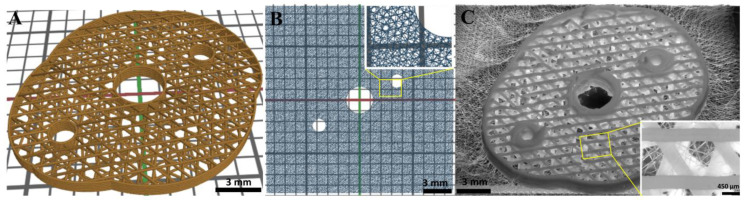
Representation of the CPC+PCL design and fabrication process with a more complex pore geometry and scaffold architecture (maximum dimensions 25.7 mm × 22.4 mm). The CPC part of the scaffold (brown) was designed with six layers and a layer-to-layer orientation of 55° (**A**). The cavities in the scaffold provide further design options, e.g., allowing implant fixation with surgical screws or the insertion of (synthetic) blood vessels through the construct. Two layers of PCL fibers (blue) were placed between each of the CPC layers with a layer-to-layer orientation of 35° to minimize deflection by the CPC strands (**B**). The insert exploits the PCL fiber geometry. The resulting scaffold is shown in (**C**) with an insert displaying the crossing of the CPC pores by the PCL fibers (scale bars: (**A**) = 3 mm, (**B**) = 3 mm, (**C**) =3 mm and 450 µm).

Based on the above observations of the deflection of the PCL fibers by the CPC strands, a scaffold with more complex morphology, pore geometry and adjusted microfiber pattern was printed (Figure 11). As designed before, two PCL fiber layers were sandwiched between each of the CPC layers (total number of CPC layers: 6). The CPC layer-to-layer orientation was chosen at 55° and that of the PCL fibers at 35°, so that the CPC pores could be bridged considerably more strongly by PCL fibers. Furthermore, it was possible to provide cavities (diameters 3 mm and 1.5 mm) in the scaffolds for possible implant fixation by surgical screws and for the potential consideration of (synthetic) blood vessels.

## 4. Discussion

In biomedical AM and biofabrication, the number of available fabrication methods, which process various material types, has been increasing drastically. Combining different techniques in one scaffolding process provides great potential for the generation of patient-specific implants and tissue models. Recent developments have drawn attention to the necessity for combinatorial multi-technological approaches in the field [37,42,43,44]. The toolbox of single-process biofabrication techniques that can be considered here is enormous, including 3D plotting or fused deposition modelling on a larger scale, and inkjet printing or MEW on a smaller scale.

Towards the fabrication of bone tissue substitutes, both PCL and CPC are highly potent biomaterials for bone regeneration and osseointegration of scaffolds. So far, no one has presented a combination of MEW-based fabrication of PCL microfibers with the 3D plotting of open-porous cement scaffolds in one alternating process that allows free choice over varying patterns and designs. With the combination of 3D plotting of the biomaterial ink CPC and the MEW of fine PCL microfibers, we present a new versatile option for the toolbox of biofabrication. This study demonstrates the benefit of this technological approach on different levels, including flexibility of architectural design of pores that impacts pore bridging behavior after scaffold seeding, as well as a stable interface and, as a result, increased integrity of the hybrid structure even after CPC strand fracture, which may prove useful in mitigating complications post-implantation.

### 4.1. Technical Versatility of the Hybrid Bioscaffolding Process

We demonstrated that PCL fibers could be integrated into CPC scaffolds in an alternating, freely adjustable pattern with high fidelity and stability, in one combined fabrication process (Figure 2). This is the first demonstration of MEW over open-porous cement scaffolds, which makes this a tailored scaffold system based on two totally different material types. For MEW, an electric field (E-field) is used to stabilize the fiber deposition by electrostatically drawing the material. Usually, a local offset between the nozzle and the fiber deposition point is defined. The extent of this lag depends on viscosity, mass flow and electric field, and can be used to additionally draw the fiber mechanically and thus, further reduce the fiber diameter [45]. To ensure the regularity of fiber deposition, a homogeneous E-field is required [29]. Only when taking into account all printing parameters (nozzle-bed distance, pressure, E-field, moving velocity), can a balance be established between the material reservoir at the nozzle tip and the deposited volume, required to ensure a constant fiber diameter. In contrast, an inhomogeneous E-field implies local differences of the field strength, which can attract or deflect the fiber from its prescribed path [35]. This leads to variations in the lag of the fiber and thus in the elongation process (so-called fiber pulsing) [40] resulting in varying fiber diameters. It can also affect the quantity of material at the nozzle, as different quantities of material are pulled from there due to the fluctuating fiber thickness. This can lead to an accumulation of material at the nozzle, which is then deposited in droplets on the existing PCL scaffold; this phenomenon is described as “long beading” effect [40].

The employed CPC had a higher conductivity compared to the glass printing bed and locally reduced the distance between fiber deposition point and nozzle due to the CPC strand height, resulting in an uneven print bed and an inhomogeneous E-field. PCL fibers accumulated at the local E-field peaks and were, therefore, placed on the CPC strands when printed in parallel in close proximity (Figure 2 and Figure 3; Supplementary Material, Figure S6). This deflection of the fibers led to fiber pulsing and a variation of the fiber diameters (min. 15 µm–max. 25 µm). The effect of long beading could be avoided by optimizing printing parameters towards a more uniform material delivery, among others by keeping the relative distance (at 5 mm) between the current CPC layer and the nozzle constant. Additional optimization could be possible by an online adjustment of the z-height of the nozzle to the individual CPC strand heights in one layer. As observed, fiber pulsing increased with the amount of CPC layers deposited in more volumetric dimensions. Therefore, irregularity of the MEW fiber pattern increased with scaffold height. Adjusting the high voltage might counteract and stabilize the jet [46]. However, considering the risk of dielectric breakdown as reported by Saidy et al. [29], a constant voltage of 6.1 kV was maintained instead. Even with these difficulties, a continuous fiber was deposited even in/on the large scaffolds with a height above 2 mm, in clinically relevant xyz dimensions. The freely adjustable hybrid fabrication process was also successfully applied to more complex CPC+PCL geometries (Figure 11). To overcome the deflections, a first test of printing at a PCL to CPC angle other than 0/90° showed a reduced level of deflection of the PCL fibers when placed onto CPC in 45° (Supplementary Material, Figure S7). However, the exact influence of the relative angle variation and the associated effects requires further investigation to ensure reproducible fiber structures.

### 4.2. Stiffness of the Hybrid Scaffolds

On a microstructural level, the effect of hydrophilization on the materials, conducted to improve the initial cell adhesion to the PCL fibers, was evaluated [47,48,49]. After 2–3 days of hardening at 37 °C in humid atmosphere, fully formed HAp crystals were not affected visually by the rather harsh alkaline treatment in 5 M NaOH (Figure 4C,F). The nanocrystalline structure closely resembles the nanostructure of fully set CPC published in earlier studies [4,5]. Those would only be expected to show adverse formation effects in an acidic environment, while NaOH concentrations might, on the contrary, lead to a more pronounced HAp formation in the case of a cement that has not yet entirely completed the setting reaction [50]. The appearance of microcracks in addition to the surface roughness [51] in the PCL fibers after etching (Figure 4E,F) might indicate an impact by strong forces that the PCL fibers experienced during hardening of the CPC strands, proving that both materials integrate with each other. Other reasons might be the electrostatic drawing towards the CPC, residual stress, or thermal contraction. On the other hand, the NaOH-mediated hydrophilization of PCL (here: 5 M, 1 h) is not expected to lead to an adverse effect on mechanical properties, as Bosworth et al. had observed a negative impact only by a NaOH treatment for more than 4 h in 10 M NaOH [48]. Furthermore, the incorporation of PCL microfibers in different CPC scaffold designs did not lead to a significantly changed stiffness (Young’s Modulus, compressive strength). With a higher CPC spacing, the mechanical properties decreased, since larger pore size resulted in a decreased surface area that the stress was applied to, with the CPC being the dominant material in compression. However, for F_1_ and E_1_ in scaffolds with smaller CPC strand distances (800 µm, 1200 µm), we observed a trend of slightly decreasing scaffold stiffness by addition of PCL microfibers in comparison to plain CPC scaffolds with same strand distances (Figure 5). This indicates that the PCL fibers placed between two CPC strands at their interface might impede the CPC to CPC layer fusion. This might impede the CPC to CPC layer fusion and therefore of the CPC to CPC interface. Nonetheless, no distinct difference between the pure CPC and the composite scaffolds was observed, and since the obtained values of Young’s modulus and compressive strength were in agreement with those obtained in previous investigations of pure CPC scaffolds, it can be concluded that the CPC structure carries the mechanical load [52,53]. Additionally, the study revealed that the overall integrity of the scaffolds during handling was markedly increased. Damaged CPC strands did not lead to a disintegrated scaffold as it was for plain CPC scaffolds and the combination enhanced the flexibility of the scaffold, as shown in Figure 6. A tearing test demonstrated that the two materials form a strong interface with the CPC, stabilizing the PCL microfibers that were integrated into the scaffold (Figure 6F). Furthermore, in the case of implantation, the surrounding PCL mesh can offer the possibility of suturing the scaffolds to the surrounding tissue for additional stabilization at the defect site.

### 4.3. Scaffold Integrity for In Vivo Application

A central advantageous aspect of the suggested system towards clinical application can be the enhanced CPC+PCL integrity during scaffold handling and in response to mechanical stress (Figure 6), such as during potential press-fit implantation [54,55]. Especially when scaffolds are applied in load-bearing conditions in small animal models, brittle tissue substitutes are sometimes difficult to handle and might be damaged pre- or post-implantation [2,56]. Scaffold residues resulting from this damage might then migrate into the surrounding tissue and induce inflammation or fibrosis that can negatively affect the tissue regeneration process [57]. Therefore, the incorporation of PCL microfibers adds a new option to the previous externally and internally applied CPC-reinforcing strategies [58], whereas the surrounding PCL frame (Figure 6C,F) might even be advantageous for fixing an implant to adjacent tissue. To avoid potential infection, incorporation of silver nanoparticles in PCL for an antimicrobial effect is also possible [59]. As demonstrated in Figure 6E,F, the dominant component in response to tension after CPC strand fracture is the PCL. In vitro and in vivo degradation behavior for both materials have been studied before. While the majority of the CPC is expected to be degraded after 6 months [60], the degradation rate of PCL is drastically lower (3–4 years) [61]. Therefore, the microfibers are expected to be present long enough before CPC degradation or resorption to hold the CPC in place after scaffold fracture.

### 4.4. Pore Bridging of Hybrid CPC+PCL Bioscaffolds by Cells In Vitro

In tissue engineering scaffolds, an open-porous adjustable design is needed to ensure sufficient in vitro-seeding or *in vivo*-infiltration by cells and potential in-growth of tissue and vessels from the surrounding tissue after implantation. The targeted bone tissue substitutes should possess or mediate a substantial amount of mineralization which is ensured by a high CPC content [60]. In general, the desired pore size for such scaffolds is expected in the range of 150–500 µm, with 60–80% interconnected pores [10,62]. Higher porosity by CPC strand distances > 2000 µm (which in this study corresponded with a maximum pore diameter of 1985 ± 134 µm) would not be favored and was not considered for the in vitro part of this study, as also demonstrated in previous pre-clinical studies on CPC [2,56]. Specific design can be necessary for different types of damaged tissue or different species [10]. In comparison to those previous studies, in the presented hybrid CPC+PCL system, two types of pores were classified: (i) the larger pores in the CPC structure that allow adjusting stiffness and are required for the efficient infiltration of cells and blood vessels after implantation; and (ii) the PCL mesh size that determined the pore bridging behavior of cells in scaffolds with large CPC pore diameters and can be used to control the pore bridging pattern, as Buenzli et al. had described a linear dependence of pore size/geometry and bridging time [33].

On day one, cells had attached to both materials rather randomly (Supplementary Material, Figures S3 and S4). Seeding efficiency was not affected by the addition of PCL fibers since the majority of the scaffold surface, and especially scaffold volume, were attributed to the larger CPC strands. Incorporation of the fine PCL microfibers did not result in a volume increase sufficient enough to improve seeding efficiency. It is expected that by further varying the microfiber mesh patterning [63], significant differences in seeding efficiency of hybrid scaffolds in comparison to plain CPC scaffolds might appear; this will be investigated in future studies.

We hypothesized that the addition of PCL microfibers would lead to an increased velocity of cell bridging kinetics. Even though the seeding efficiency was rather low on the scaffolds (5–35% for mOB, 1–24% for hMSC), cells colonized the CPC strands rather quickly and started to fill the pore space by day seven (hMSC, Figure 8) or by day 11 (mOB). In smaller CPC pores (strand distances of 800 and 1200 µm), the dynamics of filling the pores appeared more variable compared to single biomaterial studies [33,64]. Significant differences were only detected in local scaffold regions due to slightly inhomogeneous seeding pattern and, potentially, the differences in fiber size and mechanical properties of CPC and PCL. Therefore, initial qualitative evaluation revealed only little overall differences. hMSC were able to bridge the pores with the support of PCL by day 11 and without PCL microfibers by day 14 (Figure 8B), while mOB were not able to fully bridge the pores after 14 days even with the support of PCL microfibers. In these scaffolds, no significant difference comparing the cell numbers was detected after 1 or 7 days, possibly due to the rather low surface ratio that is attributed to the PCL compared to the large CPC strands. In the case of CPC_1600_+PCL scaffolds, a difference in bridging behavior appeared for both cell types after analyzing the morphological changes (hMSC, day 7 and 14, Figure 9 and Figure 10) and cell number quantification (mOB, day 7, Figure 10). Thus, 1600 µm was identified as a critical pore distance. The pores of the CPC_1600_ scaffolds could only be bridged through their combination with the PCL microfibers. Scaffold integrity was expected to be improved in all investigated scaffold types, whereas an advantage regarding pore bridging by seeded cells through the PCL addition was only created for CPC strand spacing of 1600 µm and bigger. For macro-porous scaffolds, the incorporation of the PCL fibers into CPC scaffolds is therefore expected to lead to a generally improved infiltration effect by cells in vitro and more effective guidance for tissue ingrowth in vivo. As demonstrated before, the structure of PCL meshes can also impact the differentiation pattern of seeded cells [65].

### 4.5. Multi-Technological Concepts—The Future of Biofabrication

The future value of combinatorial multi-technological approaches to the field of biofabrication [37,42,43] and to the success of tissue engineering strategies is unquestioned [66]. In our approach, the layer pattern can be selected freely by applying MEW over the 3D plotted CPC strands of an open-porous scaffold. Technically, MEW is applicable to various materials [23] and further allows an extension towards combinatorial approaches with 3D plottable hydrogels or bioinks [43,67]. This fosters ideas in the direction of previous initial approaches of utilizing MEW and PCL as reinforcing support for cell-laden hydrogels and 3D printed bioinks [28,68,69]. These concepts are still being further developed to pre-clinical application, e.g., towards fabrication of auricular cartilage substitutes [70]. Further research might even allow the incorporation of living cells into MEW-based fabrication concepts [71]. In the context of patient specific implants, anatomically shaped designs can be considered for hybrid CPC+PCL tissue substitutes in the future, with MEW over individually shaped constructs as suggested recently by Peiffer et al. [42]. The respective key challenges of the biofabrication community will be combining different techniques and material classes, as well as bioprinting approaches that include the processing of viable cells to volumetric tissue substitutes [72,73,74]. Bringing these aspects together with the presented novel approach, the toolbox of bioscaffolding and biofabrication techniques can be further extended in the future.

## 5. Conclusions

In this study, for the first time, the technical application of MEW over non-hardened, 3D plotted CPC structures has been investigated. This combination allows free choice over architecture and stacking order of CPC and PCL layers during the fabrication of hybrid bioscaffolds based on two different biomaterial inks with an approval for clinical application respectively. With this combined process for a free scaffold design in an accurate, interchangeable order, we were able to fabricate fine PCL microfibers inside open-porous CPC scaffolds resulting in a controllable pattern with a stable CPC to PCL interface. The CPC+PCL scaffolds, after alternating application of the combined methods of MEW and 3D plotting, showed an improved integrity during mechanical and manual handling, due to a strong interface ensuring the integration of two different biomaterial types. At a CPC strand distance of 1600 µm, hMSC were able to bridge the pores after 2 weeks only with the support of PCL microfibers. These CPC_1600_+PCL scaffolds presented a compressive strength of 4 MPa and a Young’s modulus of 50 MPa without an adverse effect of the PCL fibers on the mechanical properties.

Proving the capability of combining these two methods can be an important step for biomedical AM since multi-fabrication approaches will be crucial for the further development of the field. In the face of these future developments, we provided another versatile system to add to the toolbox of biomedical AM.

## Figures and Tables

**Table 1:** Summary of the applied scaffold and cell types with resulting maximum pore diameters (mean ± SD) for in vitro experiments.

In Vitro Exp.#	Scaffold Type	Cell Type (s)	Maximum CPC Pore Diameter Ø (Mean ± SD, n > 6)
**1**	CPC_800_+PCL CPC_1200_+PCL	mOB	457 ± 30 µm 685 ± 72 µm
**2**	CPC_1200_+PCL	mOB/hMSC	591 ± 82 µm
**3**	CPC_1600_+PCL	mOB/hMSC	1356 ± 191 µm

## Data Availability

All data that support the findings of this study are included within the article (and any Supplementary Information Files).

## Supplementary Materials

The following supporting information can be downloaded at: https://www.mdpi.com/article/10.3390/jfb13020075/s1, Supplementary Material File (pdf) containing: Figure S1: Representative stress–strain curve from one CPC_800_+PCL scaffold. Figure S2: Mechanical properties of CPC and CPC+PCL scaffolds with different CPC strand distance (800 µm, 1200 µm, 1600 µm, 2000 µm). Figure S3: SEM images of mOB-seeded hybrid CPC+PCL scaffolds (day 1). Figure S4: MC3T3-E1 attachment (yellow arrows) on CPC and on PCL fibers that were printed or dragged out of the scaffold plane. Figure S5: cLSM fluorescence images of hMSC cell morphology and focal adhesion points. Figure S6: Light microscopy image of 1 CPC layer + 4 PCL layers with a 90° PCL to CPC-orientation. Figure S7: Light microscopy image of 1 CPC layer + 2 PCL layers with a 45° PCL to CPC-orientation.
