# Supplementary material for: 3D Plotting of Calcium Phosphate Cement and Melt Electrowriting of Polycaprolactone Microfibers in One Scaffold: A Hybrid Additive Manufacturing Process
Source: J Funct Biomater. 2022 Jun 8;13(2):75. doi: 10.3390/jfb13020075 (PMC9225379; doi:10.3390/jfb13020075)
Supplement: Supplementary file 1 [file jfb-13-00075-s001.zip › jfb-1737558-supplementary.pdf]

# SUPPLEMENTARY MATERIAL

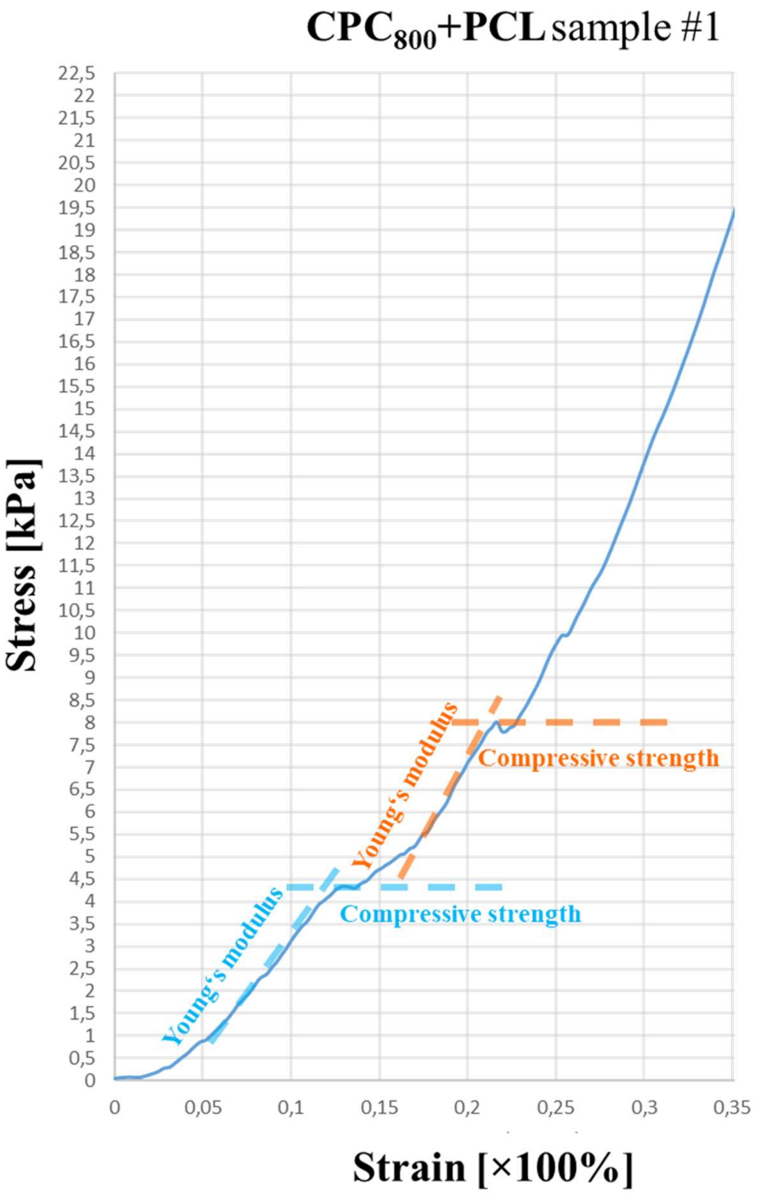


**Figure S1:** Representative stress-strain curve from one $CPC_{800}$+PCL scaffold, with the respective two breaking points for determination of compressive strength and Young's modulus

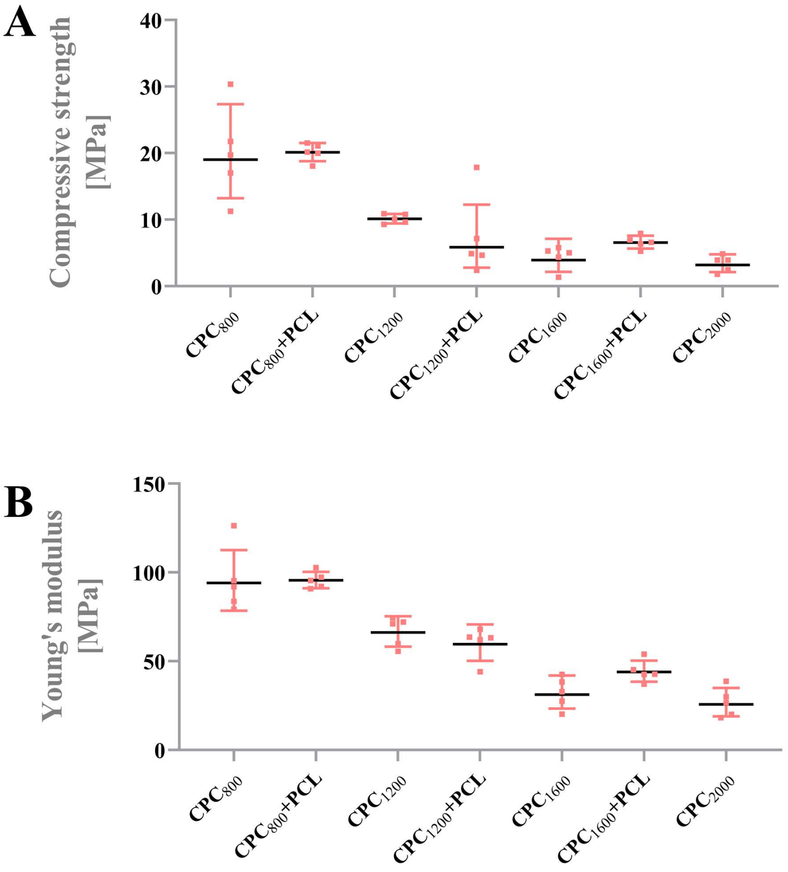


**Figure S2**: Mechanical properties of CPC and CPC+PCL scaffolds with different CPC strand distance (800 µm, 1200 µm, 1600 µm, 2000 µm), calculated at second breaking point of the stress-strain curve. **A**: Compressive strength of CPC and CPC+PCL scaffolds. n = 3. **B**: Young's modulus of CPC and CPC+PCL scaffolds. n = 5.

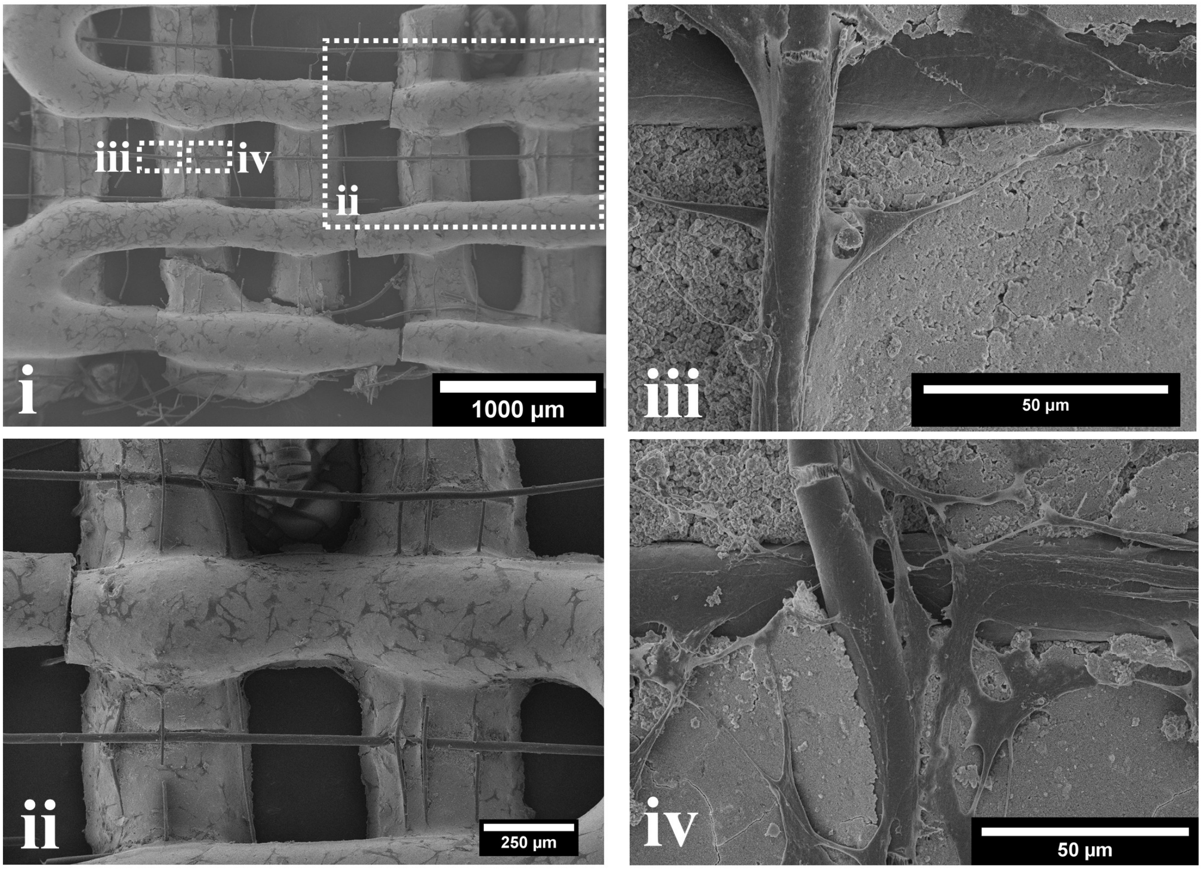


**Figure S3**: SEM images of mOB-seeded hybrid CPC+PCL scaffolds (day 1), overview image (i; scale bar = 1000 µm), higher magnification images focusing on biphasic interwoven structure (ii; scale bar = 250 µm) and cell attachment to CPC strands and PCL fibers (iii, iv; scale bar = 50 µm)

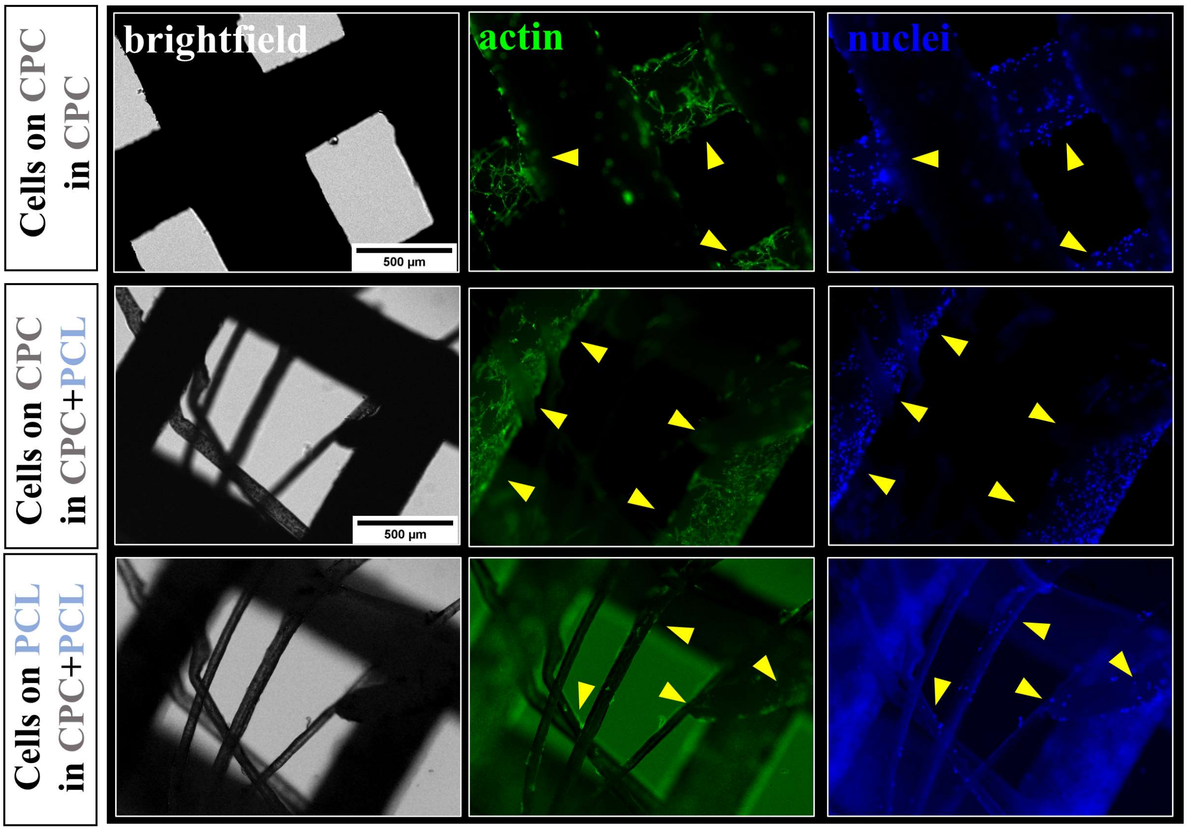


**Figure S4:** MC3T3-E1 attachment (yellow arrows) on CPC and on PCL fibers that were printed or dragged out of the scaffold plane, on day 1 after seeding to CPC in CPC scaffolds (A, upper panel), and cell attachment to both materials CPC (A, middle panel), and PCL fibers (lower panel) in CPC+PCL hybrid scaffolds.

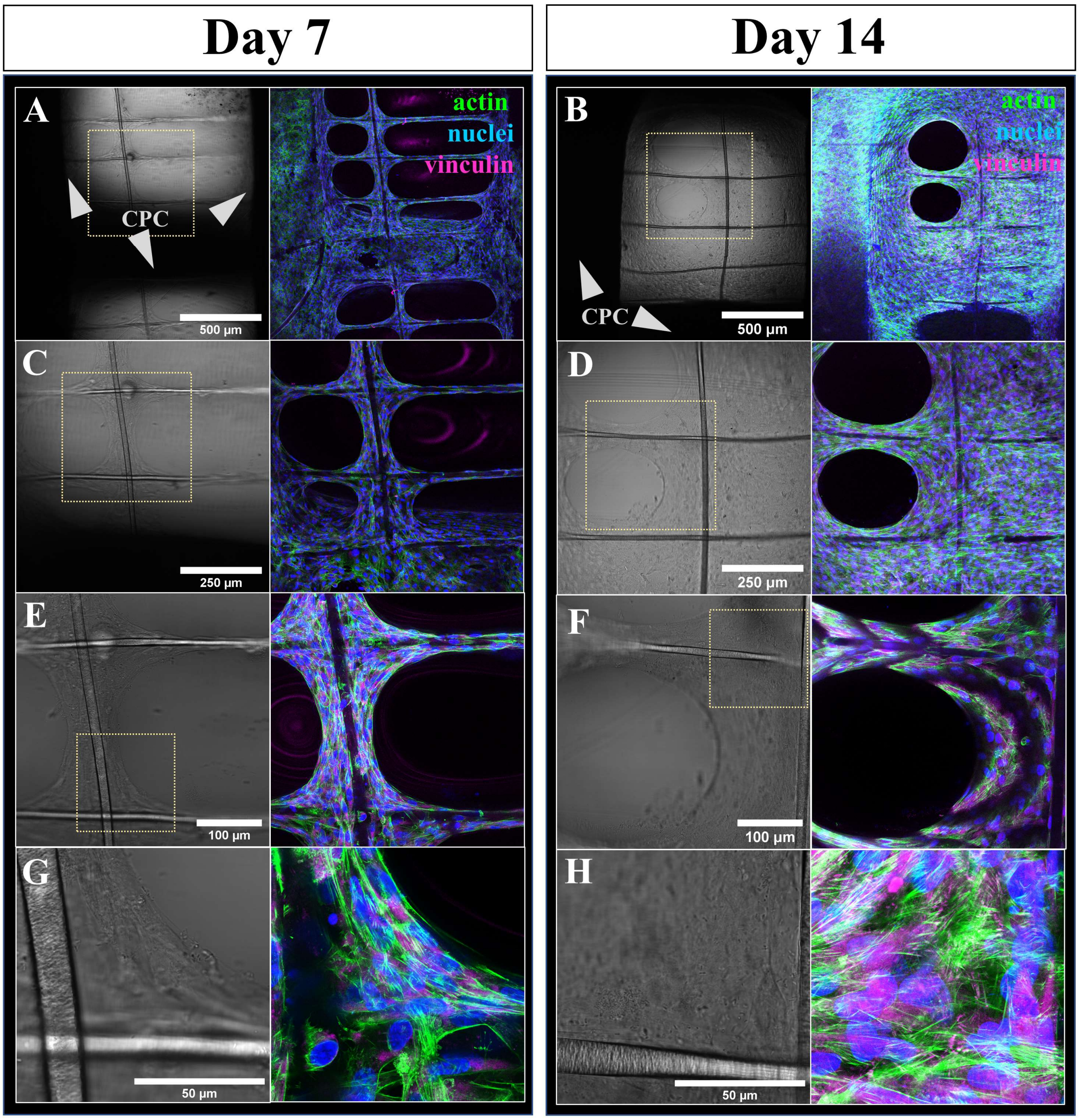


**Figure S5**: A: cLSM fluorescence images of hMSC cell morphology and focal adhesion points on day 7 and 14 of cultivation in $CPC_{1600}$+PCL scaffolds with a CPC strand distance of 1600 µm: cytoskeleton in green (phalloidin), cell nuclei in blue (DAPI), vinculin/focal adhesion points in magenta (anti-vinculin immunostaining), scale bars in A/B represent 500 µm, scale bars in C/D represent 250 µm, scale bars in E/F represent 100 µm, scale bars in G/H represent 50 µm.

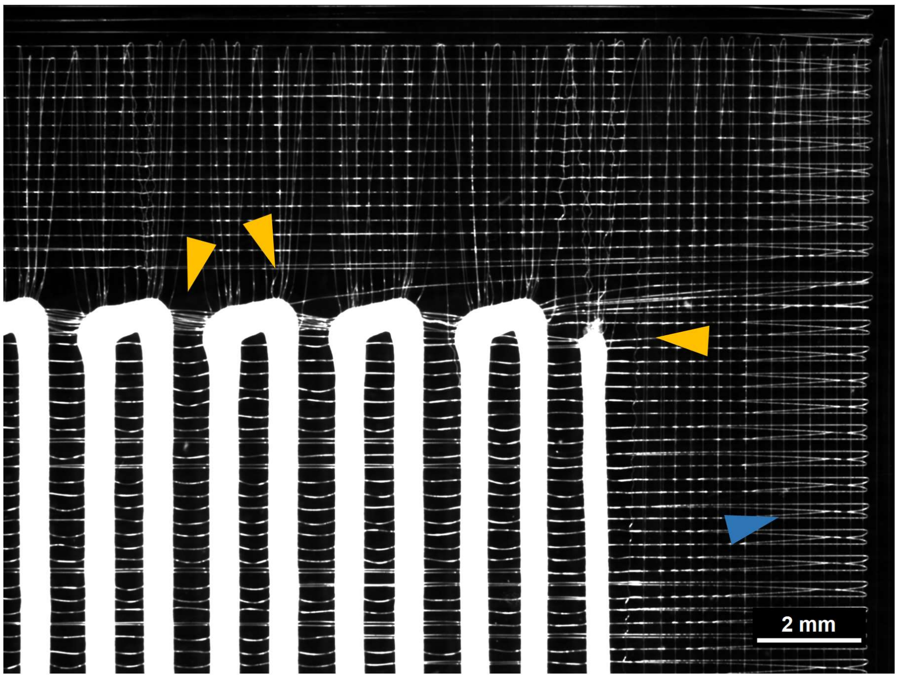


**Figure S6:** Light microscopy image of 1 CPC layer + 4 PCL layers with a 90° PCL-to-CPC-orientation. Yellow arrows indicate fiber accumulation due to a locally increased electrical field strength. Blue arrow indicates regular fiber structure at the outer edges of the PCL scaffold, scale bar = 2 mm.

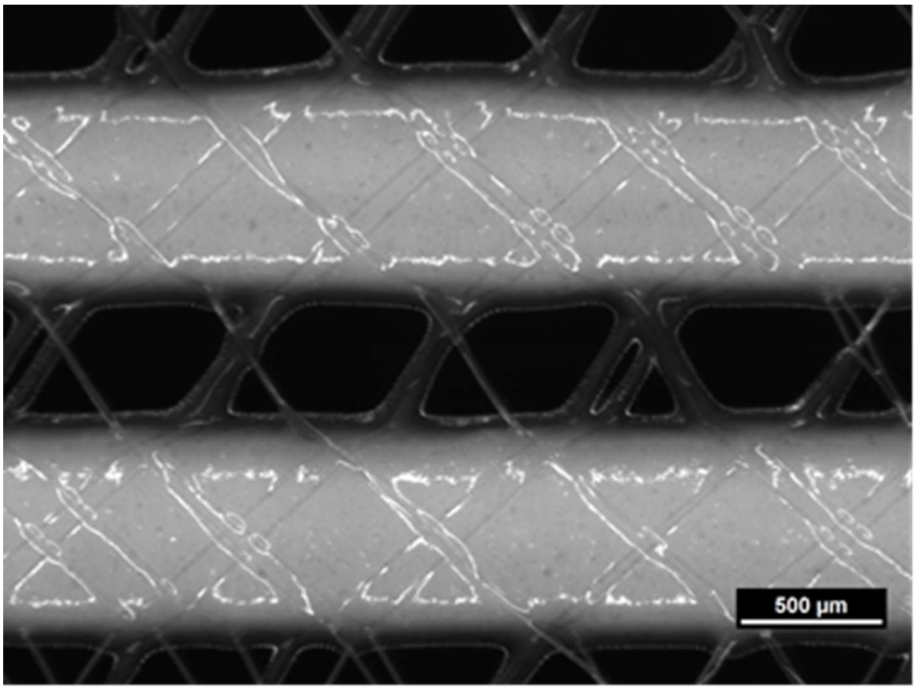


**Figure S7:** Light microscopy image of 1 CPC layer + 2 PCL layers with a 45° PCL-to-CPC-orientation to the CPC which can lead to a reduced level of deflection of the PCL fibers in comparison to PCL fibers printed in parallel to CPC strands, scale bar = 500 µm.
